# Decoding the leaf apical meristem of *Guarea glabra* Vahl (Meliaceae): insight into the evolution of indeterminate pinnate leaves

**DOI:** 10.1038/s41598-024-55882-0

**Published:** 2024-03-02

**Authors:** Yasutake Moriyama, Hiroyuki Koga, Hirokazu Tsukaya

**Affiliations:** Graduate School of Science, The University of Tokyo, 7-3-1, Hongo, Bunkyo-Ku, Tokyo, Japan

**Keywords:** Morphogenesis, Evolutionary developmental biology, Plant stem cell

## Abstract

In seed plants, growth of shoots and roots is indeterminate, while leaves are typically determinate organs that cease to grow after a certain developmental stage. This is due to the characteristics of the leaf meristem, where cell proliferation activity is retained only for a limited period. However, several plants exhibit indeterminacy in their leaves, exemplified by the pinnate compound leaves of *Guarea* and *Chisocheton* genera in the Meliaceae family. In these plants, the leaf meristem at the tip of the leaf retains meristematic activity and produces leaflets over years, resulting in a single leaf that resembles a twig. The molecular mechanism underlying the indeterminate leaf meristem of these plants has not been examined. In this research, we used *Guarea glabra* as a model to investigate the development of indeterminate pinnate leaves. Transcriptome analyses revealed that the gene expression profile in leaf apex tissue differed from that in the shoot apex. However, a class 1 KNOTTED-LIKE HOMEOBOX (KNOX1) gene which is lost in Brassicaceae was highly expressed in both tissues. We established an in situ hybridisation system for this species using Technovit 9100 to analyse the spatial expression patterns of genes. We revealed that the leaf meristematic region of *G. glabra* expresses KNOX1, *LEAFY* and *ANGUSTIFORIA3* simultaneously, suggesting the involvement of these genes in the indeterminacy of the leaf meristem.

## Introduction

Indeterminate growth is one of the most conspicuous features of plants compared with animals. Plant shoots and roots can continue to produce new organs throughout their lifetime, and this ability is achieved by the presence of meristems at the shoot and root apices^1,2^. In seed plants, shoot components are derived from shoot apical meristem (SAM). SAM maintains stem cells in an indeterminate state, and cell populations produced from stem cells eventually differentiate into various organs, including stems and leaves, or axillary shoots with a new SAM. In Arabidopsis, a typical dome-like SAM is formed during embryogenesis, and stem cells that ensure the indeterminacy of shoot growth remain in SAM. SAM formation is dependent on the expression of class I KNOTTED-LIKE HOMEOBOX (KNOX1) genes. For example, the *SHOOT MERISTEMLESS* (*STM*) gene plays an indispensable role in SAM formation since *stm* mutant seedlings display a lack or reduction in SAM^3,4^. The Arabidopsis genome encodes four KNOX1 family genes, *STM*, *KNAT1*, *KNAT2* and *KNAT6*, that redundantly contribute to maintaining meristematic activity in SAM^3,5^. In addition, a regulatory circuit including the homeobox transcription factor WUSCHEL (WUS), the peptide ligand CLAVATA3 (CLV3), its receptor CLAVATA1 (CLV1), and other components is necessary to maintain the stem cell niche in SAM^6^.

Contrary to the indeterminacy of SAM, the meristems of lateral organs, such as leaves and flower organs, generally exhibit determinate growth. As the meristems of these organs eventually lose meristematic activity, the organs cease growth after a certain period ^7^. Previous research on leaf development revealed important genes that underpin the nature of leaf meristem (LM), including *ANGUSTIFOLIA3* (*AN3*)*/GRF-INTERACTING FACTOR1* (*GIF1*). *AN3* encodes a transcriptional coactivator that promotes cell proliferation with its partner transcription factor, such as GROWTH-REGULATING FACTOR5 (GRF5) in Arabidopsis^8,9^. The *an3* mutant exhibits a drastic reduction in leaf cell number^8^. AN3 proteins are not distributed in SAM, but in leaf primordia in a manner highly correlated with meristematic activity in leaf development. In Arabidopsis, AN3 is distributed throughout young primordia, then restricted in the basal region of primordia, before finally disappearing^8,10^. A similar function is reported for an *AN3* ortholog in rice, though there are slight differences in expression patterns^11^. Therefore, in LM, meristem establishment proceeds via a mechanism distinct from that in SAM, which is characterised by transient meristematic activity, and AN3 is likely to be a part of such a mechanism.

However, some seed plants exhibit indeterminacy even in the LM, and their leaves can grow infinitely^12^. For example, the genus *Monophyllaea* and some species of the genus *Streptocarpus* in the Gesneriaeae family, termed one-leaf plants, make one of their cotyledons grow indeterminately, and do not form a new stem or a foliage leaf^13–15^. Their meristems for cotyledon growth, termed basal meristems, retain their meristematic activity for an extraordinarily long period, resulting in a huge cotyledon. *Welwitschia mirabilis* is a gymnosperm species that retains two indeterminate foliage leaves. It forms ribbon-like leaves and maintains continuous LM activity at the base^16^. In some of these cases, involvement of the KNOX1 gene has been suggested. In the simple leaf development of a typical seed plant, KNOX1 gene expression is restricted in SAM and is not observed in leaf primordia^17^. However, in *Streptocarpus*, expression of an STM ortholog in the basal meristem was detected^18^. Similarly, KNOX1 expression was detected in the basal meristem of leaves, as well as SAM, in *W. mirabilis*^19,20^. These results suggest that the unusual expression of the KNOX1 gene in LM of these plants contributes to its extraordinarily long proliferation activity. Meanwhile, in *Monophyllaea*, *MgSTM* expression was not detected at the basal meristem, suggesting that the mechanisms for indeterminate LM activity are not the same among lineages^21^.

**Figure 1:**
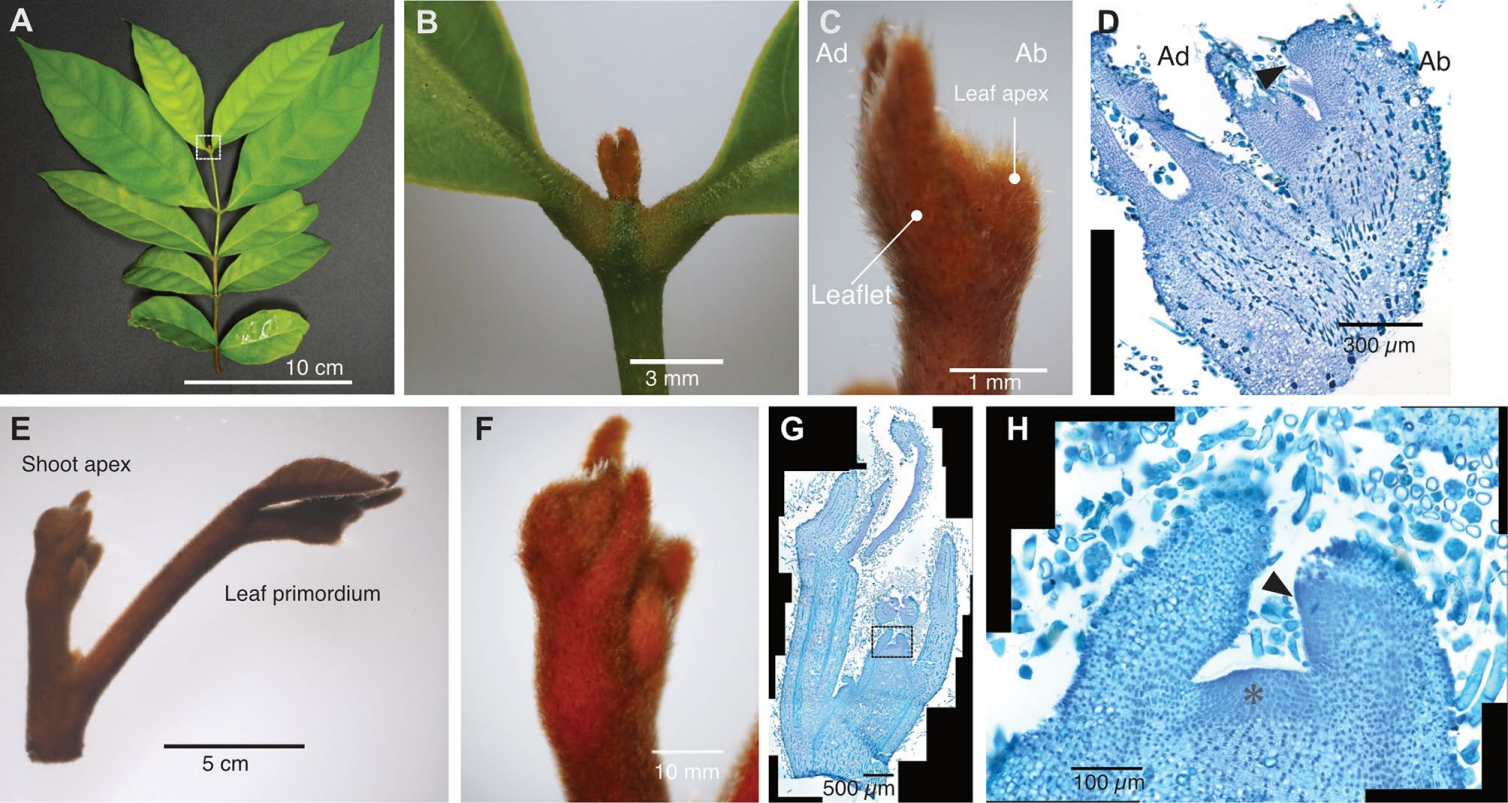
Morphology of indeterminate compound leaf and a shoot apex of *G. glabra*. (**A**) Whole leaf shape. (**B**,**C**) Magnified images of the leaf apex (LA) indicated by a square in panel (**A**). (**B**) Adaxial view. (**C**) Lateral view. (**D**) Longitudinal section of LA. The arrowhead indicates leaf apical meristem (LAM). (**E**) Shoot tip of *G. glabra*. (**F**) Magnified image of a shoot apex (SA). (**G**) Longitudinal section of SA. (**H**) Magnified image of shoot apical meristem (asterisk) and leaf primordia. The arrowhead indicates LAM. *Ad* adaxial side, *Ab* Abaxial side.

Members of the genus *Guarea* (Meliaceae) also have indeterminate leaves^22^. Unlike the aforementioned herbal plants with indeterminate simple leaves, *Guarea* is a tree genus possessing pinnate compound leaves (Fig. 1A). A *Guarea* leaf never produces a terminal leaflet, and the leaf apex keeps proliferating and forming new pairs of leaflets over many years, like a twig. A similar leaf feature is also reported in the genus *Chisocheton,* which belongs to the same family as *Guarea*^23^. Previous anatomical observations in *Guarea* and *Chitocheton* revealed that the leaf apices of these plants maintain meristematic tissue named leaf apical meristem (LAM)^22–24^. Phylogenetic analysis of Meliaceae suggests that the indeterminate leaf traits have evolved independently in *Guarea* and *Chisocheton* lineages since they are not sistered^25^. Despite the morphological and anatomical evidence, the molecular characteristics of the indeterminacy of LAM in this type of indeterminate leaf remain unexplored. Here, we explored the molecular basis of LAM in indeterminate compound leaves of *Guarea* species, using *Guarea glabra* Vahl as a model.

## Results

### Morphological analysis of *Guarea glabra* LAM

Previous studies reported that the indeterminate compound leaves of *Guarea* and *Chisocheton* have a LAM at the tip^22–24^. However, the precise position and size of LAM have not been reported for *G. glabra*, hence we compared the structures of the leaf and shoot apices (Fig. 1, Supplemental Figs. S1 and S2). *G. glabra* leaves have a bud-like structure at the apex, comprised of several pairs of leaflet primordia (LP) and a leaf apex (Fig. 1A –C). In the longitudinal section of the leaf apex, we observed tissue consisting of small cells at the very tip of the apex, presumed to be LAM (Fig. 1D). This undifferentiated tissue, which was identified by smaller cell size, and richer in cytoplasm stained by Toluidin Blue O, than other tissues, was not clearly divided from the youngest LP. Thus, LAM and the earliest stage of LP share the undifferentiated cell population, and they were barely distinguishable. In the shoot apex we observed SAM, with a typical dome-like structure and undifferentiated small cells with rich cytoplasm surrounded by protrusions of leaf primordia (Fig. 1G,H). Compared with SAM, LAM has a clear dorsiventrality and does not have axial buds or meristems, indicating that the indeterminate leaves of *G. glabra* are indeed single compound leaves.

### Transcriptome analyses reveal KNOX1 gene expression in LAM

**Figure 2:**
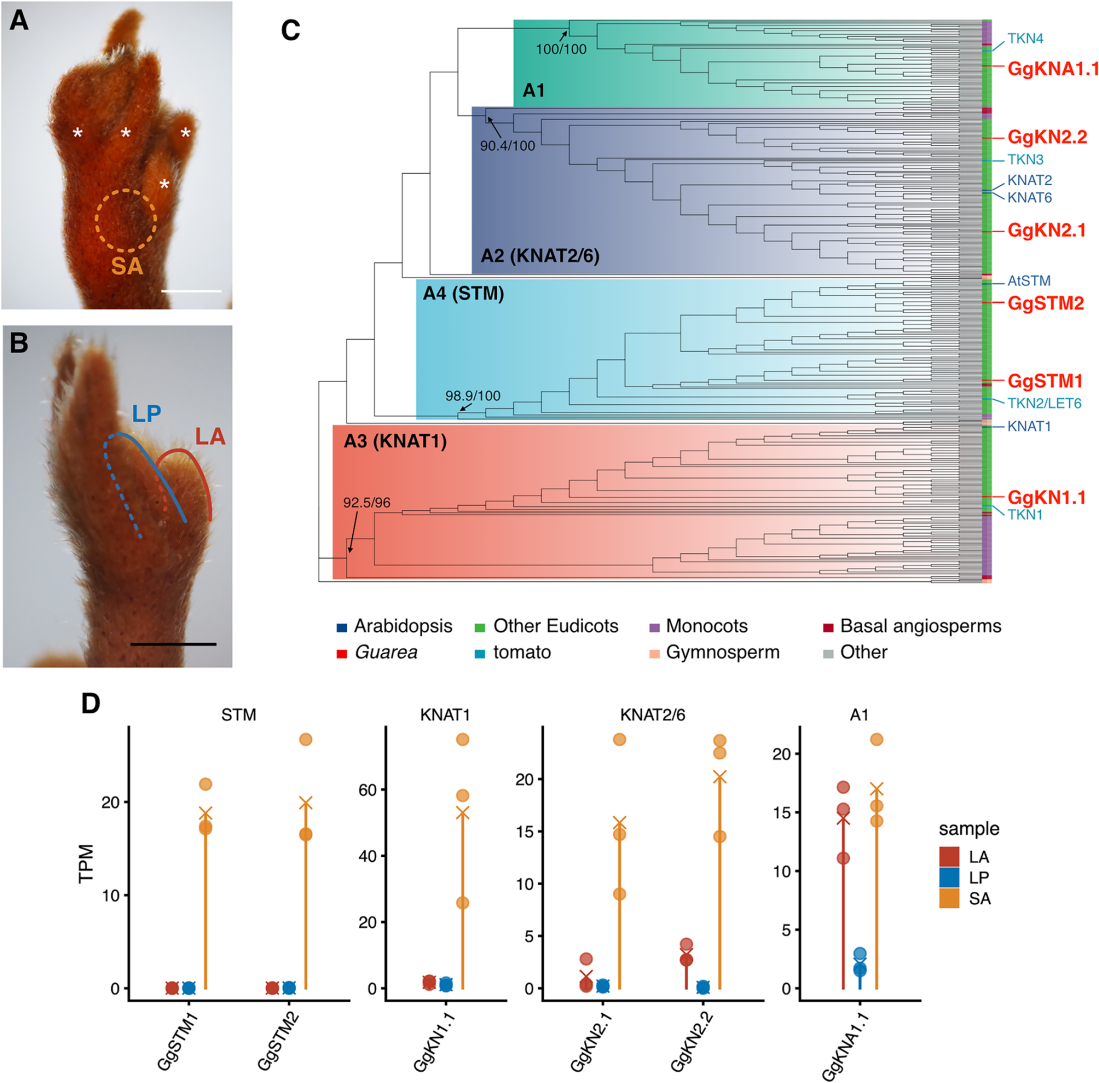
RNA-seq analysis of leaf tissues and class I KNOX genes in *G. glabra*. (**A**,**B**) Tissues collected for RNA-seq. (**A**) Shoot apex (SA) tissue was corrected after the removal of leaves or leaflet primordia denoted by asterisks. (**B**) Leaf apex (LA) and leaflet primordia (LP) were collected from the tips of well-grown leaves. (**C**) Maximum likelihood tree of KNOX1 protein sequences. Only major operation taxonomic units (OTUs) are denoted. Tip colours indicate species or clades of OTUs. Values on the basal nodes for subfamilies represent confidence values of Shimodaira–Hasegawa-like approximate likelihood ratio test (SH-aLRT) support (%)/ultrafast bootstrap (UFboot) support (%). The corresponding complete tree is shown in Supplementary Fig. S3. (**D**) Gene expression patterns of *G. glabra* KNOX1 genes. Transcripts per million reads (TPM) values from the RNA-seq data are shown. Each circle shows data from a sample, and crosses show the mean values of replicates.

To characterise SAM and LAM of *G. glabra*, RNA sequencing (RNA-seq) was conducted using shoot apex tissue including SAM (SA), leaf apex including LAM (LA), and leaflet primordia (LP) (Fig. 2A,B). The transcriptome sequence was assembled using paired-end RNA-seq reads from a mixture of all samples (Table 1), and expression was quantified by mapping single-read sequences from each sample to the transcriptome assembly (Supplementary Table S1).

**Table 1:** Statistics of de novo transcriptome assembly.

Total length	58,104,119
Number of putative genes	86,719
Number of contigs	142,136
N50	1050
BUSCO single copy	78.8% (1,832)
BUSCO duplicated	3.7% (87)
BUSCO fragmented	5.1% (118)
BUSCO missing	12.4% (289)

Previous work on model plants confirmed that expression of KNOX1 genes in SAM plays an important role in its indeterminacy, and that in some species with indeterminate leaves, a KNOX1 gene is expressed in LM^18–20^. Accordingly, we focused on KNOX1 family genes of *G. glabra*. Using proteomic data obtained herein, a large-scale phylogenetic tree reconstruction was conducted for KNOX1 family proteins (Fig. 2C, Supplementary Fig. S3). This analysis identified six genes belonging to four KNOX1 subfamilies from the *G. glabra* transcriptome. In addition to the three subfamilies corresponding to Arabidopsis STM, KNAT1 and KNAT2/KNAT6, we identified another subfamily lacking an Arabidopsis ortholog. This subfamily was previously denoted as angiosperm A1 clade^26^, and it contains four domains that stand for KNOX proteins (Supplementary Fig. S4). Because this A1 subfamily is broadly conserved in monocots and eudicots except for Brassicaceae, it appears to have evolved in early angiosperms, then been lost in some lineages. The *G. glabra* transcriptome included genes belonging to all four subfamilies with complete coding sequences, and we named them *GgSTM1* and *GgSTM2* (orthologs of *STM*), *GgKN1* (*KNAT1*), *GgKN2.1*, *GgKN2.2* (*KNAT2/6*) and *GgKNA1.1* (angiosperm A1). Although there may be more KNOX1 genes, since several fragmented assembled sequences showed similarity to KNOX1, we ignored these ambiguous sequences in the present analyses. Next, we examined the expression patterns of the identified KNOX1 genes (Fig. 2D). As expected, all KNOX1 genes were highly expressed in SA and showed almost no expression in LP. However, the expression level of *GgKNA1.1* in LA was comparable to that in SA, suggesting *GgKNA1.1* is active in LAM and/or very young LP (Fig. 2D).

**Figure 3:**
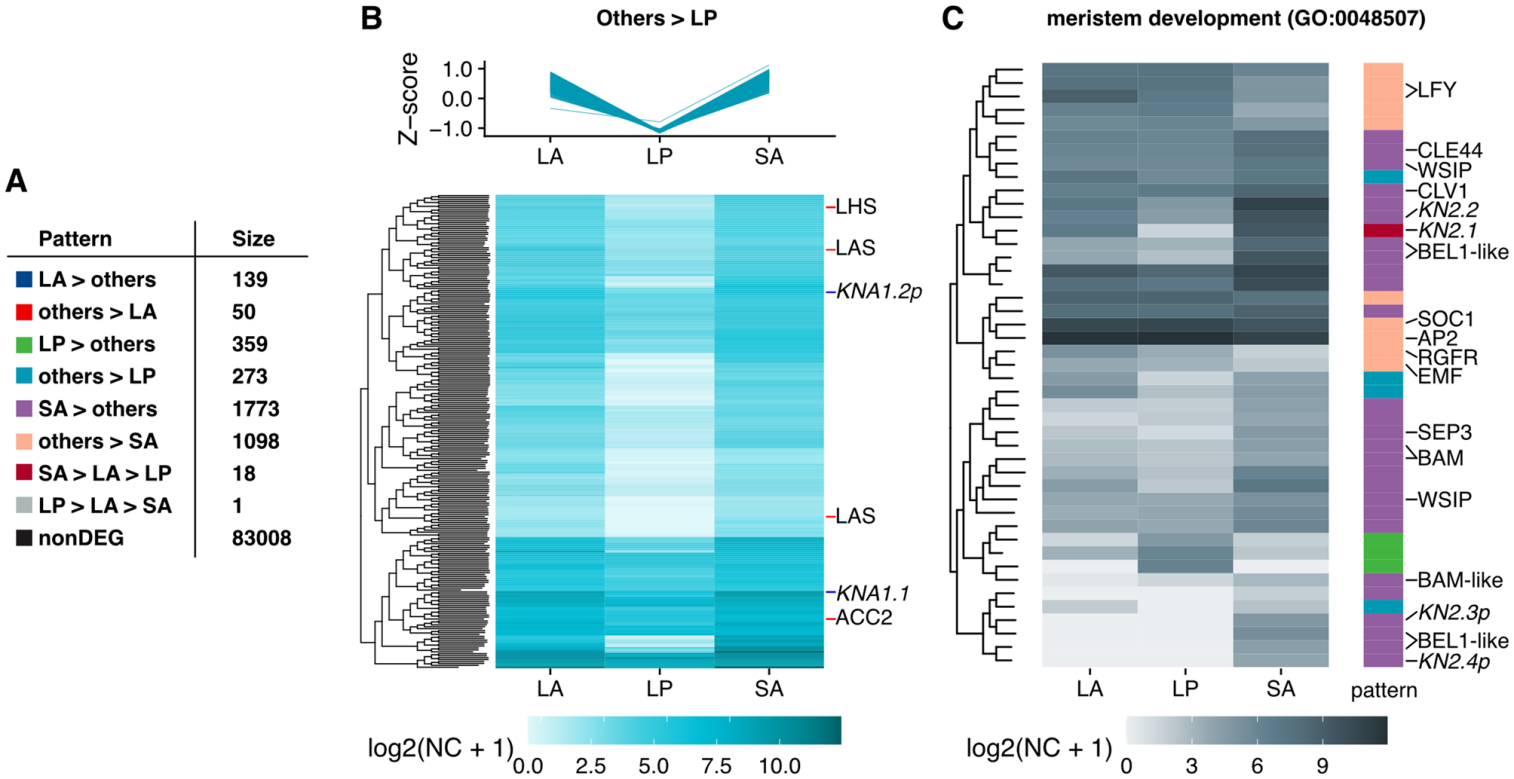
Expression patterns of genes co-expressed with *GgKNA1.1* and genes related to meristem development. (**A**) Examined expression patterns and assigned gene numbers. (**B**) Gene expression patterns of others > LP. Z-scores of normalized count (NC; top) and log2 transformed NC + 1 values (bottom) are shown. Genes in which the gene ontology (GO) term ‘meristem development’ (GO: 0048507) and KNOX A1 genes are denoted. (**C**) Expression patterns of differentially expressed genes with the GO term ‘meristem development’. The colour code for the expression pattern corresponds with panel (**A**). KNOX1 genes with the postfix ‘p’ indicate contigs with partial coding that share high similarity with KNOX1.

Based on the notable expression pattern of KNOX1 gene subfamily *GgKNA1*, we further explored genes displaying similar expression patterns, specifically those with higher expression in tissues with indeterminate meristems (LA and SA) but low expression in LP. We set up models of gene expression patterns a priori, and the posterior probability (PP) of each pattern was calculated for each gene using baySeq^27^. This resulted in 273 genes, including *GgKNA1* genes, being assigned to the pattern of interest (others > LP; Fig. 3A,B). This gene set included a few genes related to meristem development (GO:0048507), including *LATERAL SUPPRESSOR* (*LAS*) orthologs.

Other meristem-related genes that exhibited differences in expression among samples mostly belonged to a cluster with patterns in which SA expression was higher than that in other tissues (SA > others; Fig. 3C). Genes encoding BELL1-like homeobox, known to interact with KNOX1 proteins, and CLV1 and its homologs BARELY ANY MERISTEM (BAM, BAM-like), a CLV3-like peptide (CLV3/EMBRYO SURROUNDING REGION-related, CLE), and WUS-interacting protein (WSIP) showed this expression pattern (Fig. 3C). Some meristem-related genes showed lower expression in SA than in leaf tissues. Genes displaying this pattern (Fig. 3C) included APTELA2 (AP2), MADS-box protein SUPPRESSOR OF CONSTANS OVEREXPRESSION 1 (SOC1) and LEAFY (LFY). These genes are typically associated with floral meristem development^28^, but the homolog of EMBRYONIC FLOWER (EMF) that inhibits flower development also exhibited the same pattern (Fig. 3C)^29,30^.

We investigated genes showing LA-specific expression patterns (LA > others and others > LA; Supplemental Data 1 and 2), but we could not find notable genes or features based on current knowledge. Unexpectedly, LA was not similar to SA in terms of transcriptome profile despite the fact they share indeterminant meristems. Thus, it is conceivable that the acquisition of indeterminacy in LAM is not the simple implementation of the corresponding system in SAM. Expression of a few factors, such as certain KNOX1 genes or other unknown factors, may be attributed to the maintenance of LAM.

### Establishment of an in situ hybridisation method for LA tissues of *G. glabra*

Bulk RNA-seq analysis as employed above is a simple and time-effective method for comparing gene expression levels in different tissues. However, due to the complex microstructure of the leaf and shoot apices, it was not possible to perfectly separate LAM, LP and SAM. The samples we analysed by RNA-seq did not consist exclusively of LAM and SAM. Therefore, to analyse gene expression specifically in LAM we attempted in situ hybridisation (ISH), which can visualise gene expression sites in detail. In plant tissues, section ISH is generally performed by embedding the sample in paraffin. However, possibly due to the high density of hard hairs covering young tissues (Fig. 1), we were unable to make sections from paraffin-embedded samples despite trying various conditions. We then applied a sectioning method using Technovit 9100 resin, which is harder than paraffin and applicable to ISH^31^, and succeeded in sectioning.

**Figure 4:**
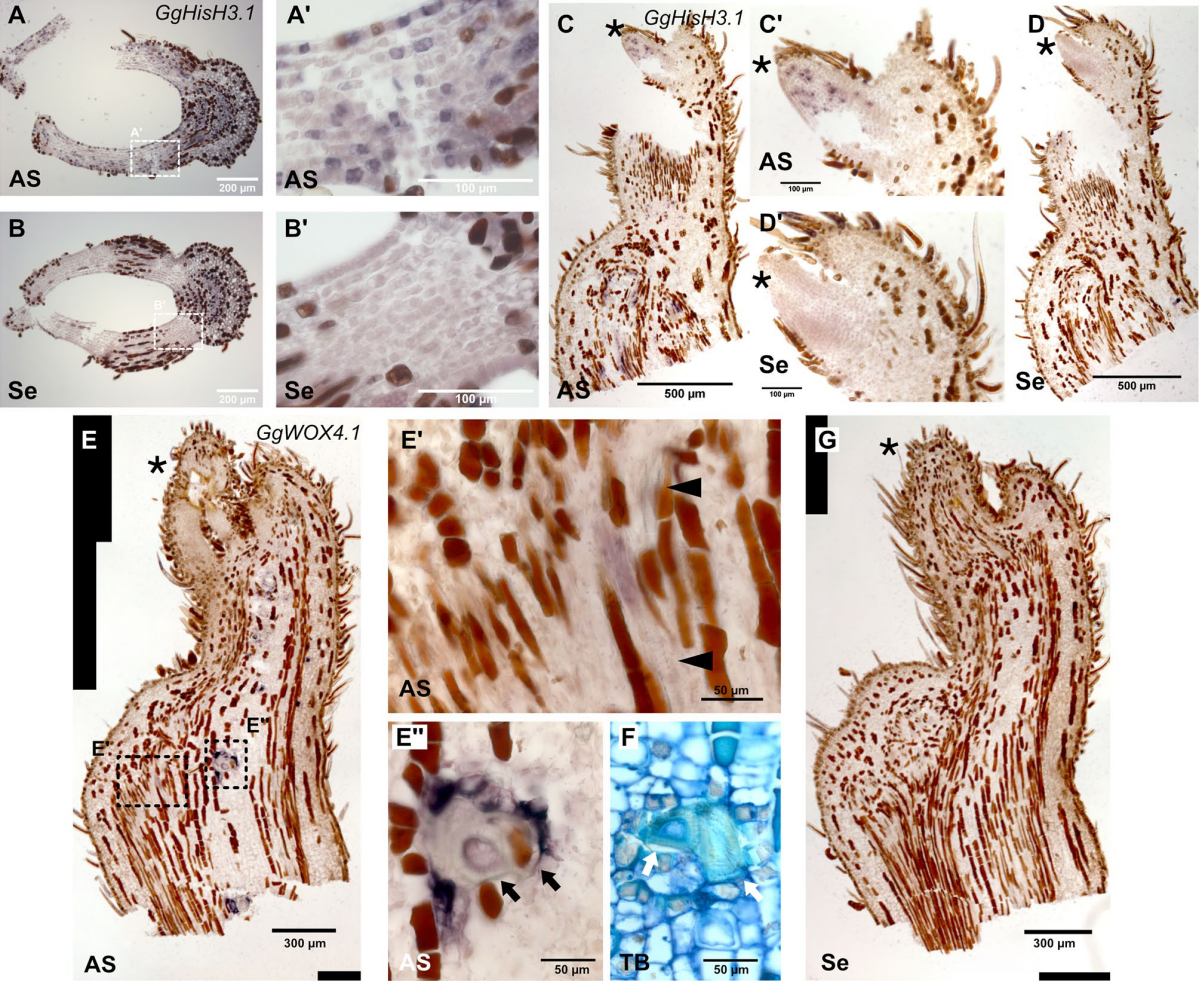
In situ hybridisation of *GgHisH3* and *GgWOX4*. (**A**–**D**) Expression patterns of the Histone H3 gene (*GgHisH3*) examined by in situ hybridisation. (**A**,**B**) Transverse section of a leaf apex at the level of comparatively large leaflet primordia. (**A**) Antisense probe and (**B**) sense probe. (**A′**, **B′**) Magnified images of the regions indicated as dotted rectangles in panels (**A**) and (**B**), respectively. (**C**,**D**) Longitudinal sections of a leaf apex. (**C**) Antisense probe and (**D**) sense probe. (**C′**,**D′**) Magnified images of leaflets in panels (**C**) and (**D**). (**E**,**G**) Expression patterns of the *GgWOX4* gene examined by in situ hybridisation. (**E**) Antisense probe and (**G**) sense probe. (**E′**,**E″**) Magnified images of staining indicated as dotted rectangles in panel (**E**). (**F**) Toluidine Blue O staining of the corresponding tissue of panel (**E″**). Asterisks indicate LP. Arrowheads indicate vessels in which helical secondary cell walls are observed. Arrows indicate presumed sclerenchyma cells with thick secondary cell walls. *AS* anti sense results, *Se* sense result (negative control), *TB* Toluidine Blue O staining.

First, we conducted ISH with Technovit 9100 sections using the probe for a Histone H3.1 ortholog (*GgHisH3.1*) to test whether the ISH protocol worked properly for *G. glabra* tissue. We identified two genes with identical amino acid sequences to Arabidopsis Histone H3.1 from the *G. glabra* transcriptome, and cloned one with higher expression. We found that idioblasts and hairs inevitably stained a brownish colour during ISH. However, clear purple signals of presumable *GgHisH3.1* expression were also detected when an anti-sense probe was used (Fig. 4A–D). The signals were observed in a ‘salt-and-pepper-like’ manner in young LP (Fig. 4A,A′,C,C′), which is typical for cell cycle-related genes^32,33^. Because histone gene expression was expected specifically in cells under S phase of the cell cycle^34,35^, the observed pattern suggested that expression of the *GgHisH3.1* gene was successfully detected by this method. We also examined *WOX4* ortholog gene expression because its spatial expression pattern was expected to differ from that of the *GgHisH3.1* gene. In Arabidopsis, *WOX4* is expressed in procambial cells and promotes vascular tissue development^36^, and thus is predicted to be expressed in vascular tissues in *G. glabra*. We identified two orthologs of *WOX4* and cloned one (*GgWOX4.1*; Supplementary Fig. S5). ISH of *GgWOX4.1* in LA tissues showed staining of vascular cells beside vessels (Fig. 4E,E′). In addition, staining was observed in cells around sclerenchyma cells with a thick secondary cell wall (Fig. 4E,E″,F). These signals were not apparent with sense probe (Fig. 4G). These results indicate that our ISH protocol with Technovit 9100 sections could detect various expression patterns in LA tissues of *G. glabra*.

### ISH of *GgKNA1*, *GgLFY* and *GgAN3* genes in LA

**Figure 5:**
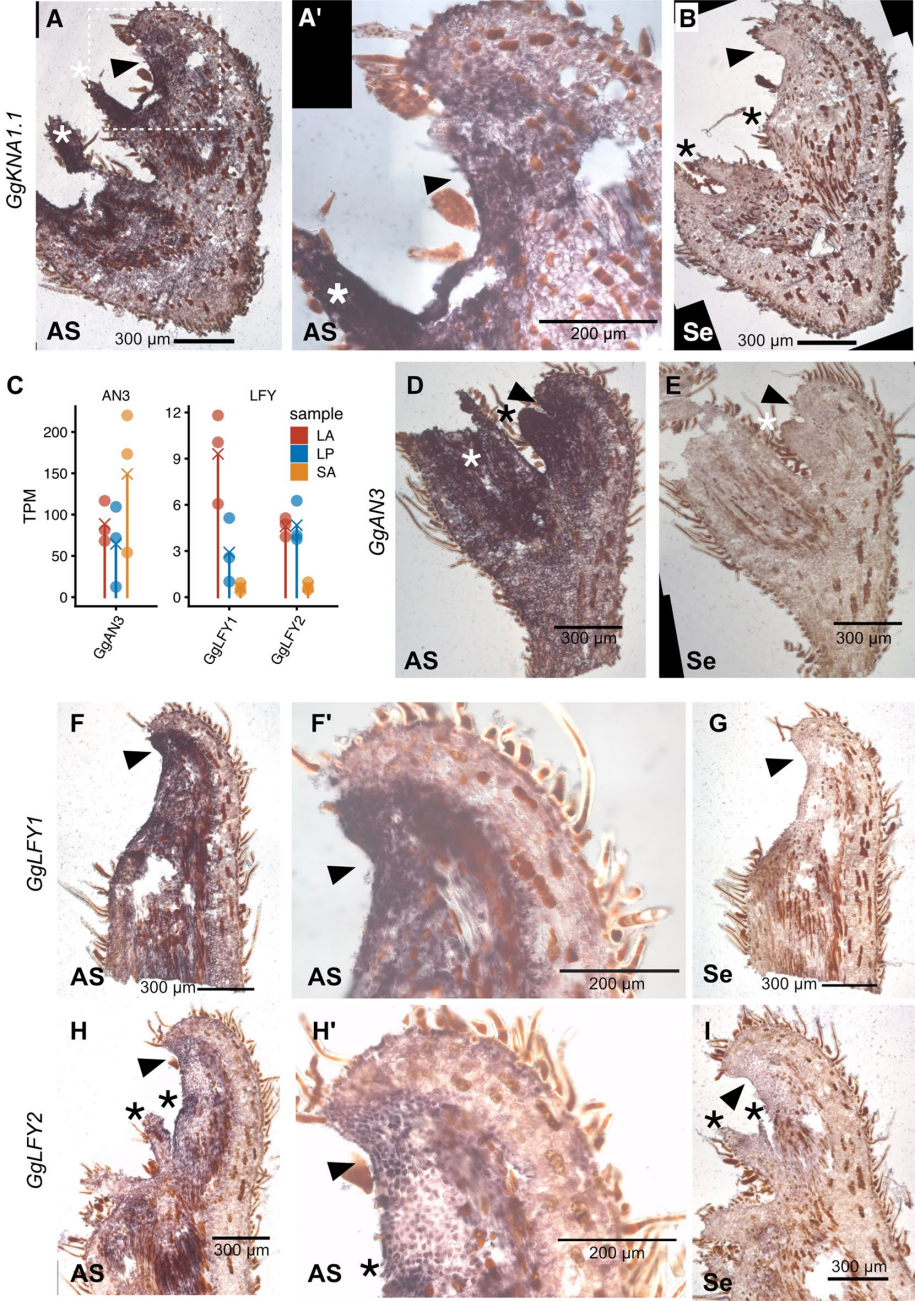
In situ hybridisation of *GgKNA1.1*, *GgAN3* and *GgLFY* in LA. (**A**,**B**) ISH of *GgKNA1.1* for LA tissue sections. (**A**) Antisense probe, (**A′**) magnified image of A indicated by the rectangle, and (**B**) sense probe. (**C**) Expression levels of *GgAN3* and *GgLFY* genes. TPM values from RNA-seq data are shown. (**D**,**E**) ISH of *GgAN3* for LA tissue sections. (**D**) Antisense probe, (**D′**) magnified image of LAM, and (**E**) sense probe. (**F**–**I**) ISH of *GgLFY1* and *GgLFY2* in LA tissue sections. (**F**,**H**) Antisense probe, (**F′**,**H′**) magnified image of LAM, and (**G**,**I**) sense probe. Arrowheads indicate LAM and asterisks indicate LP. *AS* anti sense results, *Se* sense result (negative control).

We performed ISH for genes of interest based on the results of RNA-seq and previous work (Fig. 5). First, we focused on the KNOX1 gene *GgKNA1.1* that was expressed in LA in addition to SA. The signal was clearly detected in the LAM region but only with the anti-sense probe, indicating that this gene is expressed in LAM. The signal was also detected in young LP regions (Fig. 5A,A′,B). Considering that the LA samples of RNAseq included young LPs, as well as LAM, these expression patterns were consistent with the RNAseq results (Fig. 2C). Next, we performed ISH for *GgAN3*, an AN3 ortholog (Supplementary Fig. S6), to evaluate whether LAM and surrounding tissues have leaf characteristics. In Arabidopsis, AN3 gene expression was detected in LP but not SAM^8^. RNA-seq data revealed AN3 expression in all examined tissues, including SA (Fig. 5C), but this could be because all tissues include young leaves or LP. In *G. glabra* LA, we detected signals for *GgAN3* in the broad adaxial region, including both LAM and LP (Fig. 5D,E). Finally, ISH for *GgLFY* genes was conducted, revealing expression in LA and LP according to RNA-seq data (Fig. 5C). We cloned two *LFY* paralogs (Supplementary Fig. S7) and conducted ISH in LA tissues, and *LFY* signals were detected in the abaxial region of LA, including LAM (Fig. 5F–I).

## Discussion

In this study, we explored the molecular bases of the indeterminate pinnate leaves of *G. glabra*. Because it is a tree species, several difficulties arose when attempting to apply basic molecular biological techniques typically used in model herbal species. Initially, RNA extraction failed with standard extraction protocols, possibly due to the presence of phenolic compounds. We discovered that incorporating a magnetic bead purification step dramatically improved RNA quality, which was sufficient for RNA-seq (see “Materials and methods” for details). Additionally, we encountered difficulties with paraffin sectioning, a technique commonly used for various spatiotemporal gene expression analyses. To overcome this obstacle, we developed an ISH protocol for this species using Technovit 9100. This method may be particularly effective for tissues densely covered with hard hairs, and for tree specimens that possess hard, lignified tissue, which is not suitable for paraffin sectioning.

We confirmed that KNOX1 family gene *GgKNA1.1* was expressed in LAM, and found that *GgKNA1.1* belongs to a subfamily named A1. There are no KNOX1 A1 orthologs in the genomes of Arabidopsis or other Brassicaceae species. However, this ortholog is present in *Carica papaya* (Caricaceae, Brassicales), as well as in various eudicots and monocots, indicating its specific loss in lineages leading to Brassicaceae (Supplemental Fig. S3). Due to the lack of the Arabidopsis ortholog, the function of this subfamily has not yet been as thoroughly examined as it has for other subfamilies. In tomato, expression of the ortholog *TKN4* was mainly detected in fruits, flowers and meristems, but expression was relatively low in leaves^37–39^, indicating that the gene is not active in leaves. In rice, there are two orthologs in the A1 subfamily: *OSH6* and *KN2/OSH71*. These genes were reportedly expressed in SAM and inflorescence meristems rather than leaves^40^. Although the available data is currently limited, these observations suggest that KNOX A1 is not usually active in leaves in angiosperms.

Expression of *GgKNA1.1* was also detected in young LP by ISH, consistent with the KNOX1 expression pattern in the compound leaves of tomato. In the development of a tomato leaf, *TKN1* (A3/KN1 subfamily) and *LET6/TKN2* (A4/STM subfamily) genes are expressed in primordial leaflets^41,42^, enhancing cell proliferation by activating cytokinin signalling^43–45^. In *G. glabra*, a different subfamily gene, *GgKNA1.1*, may function for the early outgrowth of leaflets in the formation of complex leaves. However, expression of *GgKNA1.1* in LP may eventually be attenuated because a dissectible size of LP used in RNA-seq exhibited low expression levels. Collectively, the results indicate that expression of an KNOX1 A1 ortholog in leaves may be a specific feature of *G. glabra*. Although a proportion of *GgKNA1.1* gene expression may be related to compound leaf formation, as in tomato, it is still a unique feature of *G. glabra* that its expression is largely restricted to the LAM region, where indeterminacy of leaves is retained. The phenomenon is similar to other species in which indeterminate leaves is associated with KNOX1 expression, such as *Streptocarpus* and *Welwitschia*^18,19^*.*

In addition to KNOX1, we confirmed that *GgLFY* showed higher expression in leaves than in SA, and expression was detected in LAM. Expression of *GgLFY* in LAM of indeterminate leaves is seemingly at odds with the fact that LFY functions in flower development, a determinate organ, in Arabidopsis and other angiosperms^46–48^. However, LFY is also known to play a role in meristem maintenance and growth, even in the vegetative phase, as a presumably ancestral function from basal land plants^49,50^. LFY orthologs are reportedly involved in SAM development in several eudicots^51,52^, and in inflorescence meristem maintenance in rice^53^. In addition, LFY orthologs are involved in compound leaf development instead of KNOX1 genes in legume species^54–57^. Therefore, in *G. glabra*, it is possible that *GgLFY*, accompanied by other floral developmental genes as a module, is involved in maintenance of meristem activity in LAM, as well as compound leaf formation.

**Figure 6:**
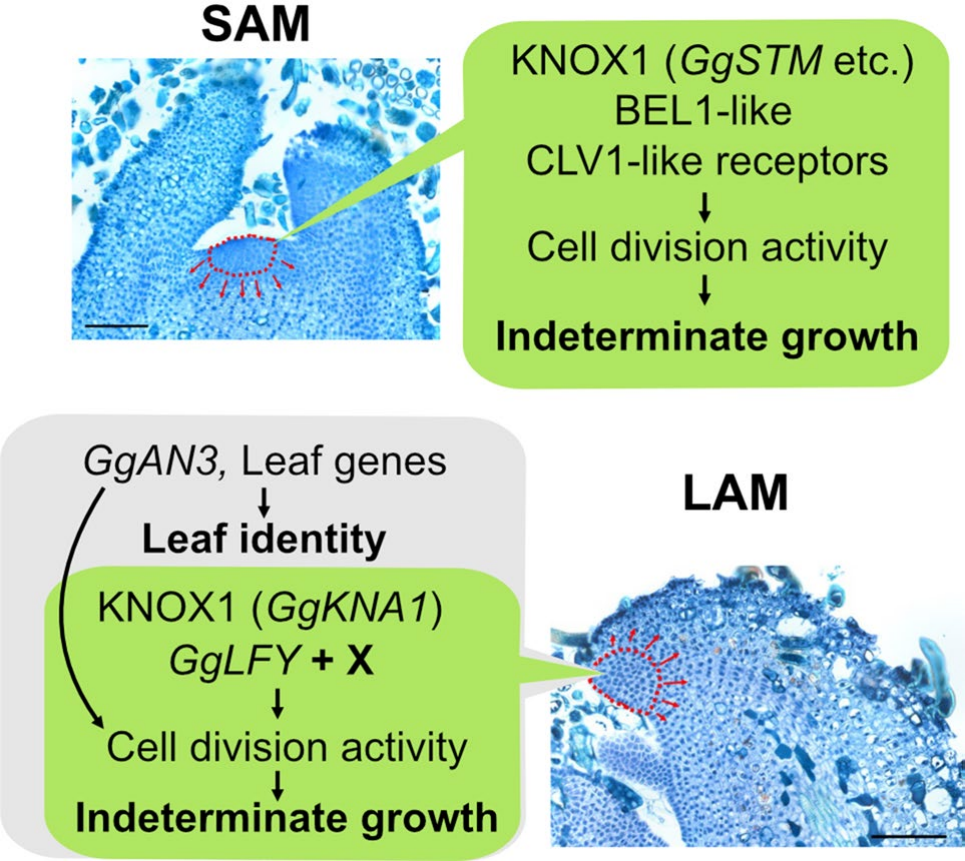
Emerging hypothesis of the mechanism of LAM indeterminacy in *G. glabra*. In SAM, a typical regulatory module for maintenance of the stem cell niche functions for indeterminate growth (top). In LAM, in addition to genes related to leaf meristem, such as AN3, expression of a few genes that function in meristem maintenance, such as A1 KNOX1 and *LFY*, provide indeterminacy of LAM (bottom). Additional unknown factors (X) are also presumed.

We also detected *GgAN3* expression in LAM and LP. Because AN3 promotes cell proliferation in leaves of Arabidopsis^8^, the ortholog was expected to be expressed in LP, presumably under the cell proliferation phase. However, expression in LAM contrasts with previous observation that AN3 was not expressed in SAM^58^. Although further functional analyses are required, we speculated that expression of AN3 may represent the identity of LAM as leaf meristem, while expression of KNOX1 and LFY provides shoot meristem-like features to LAM, resulting in indeterminate leaves (Fig. 6). Apparently, expression of only *GgKNA1.1* and *GgLFY1/2* in the LAM region cannot explain indeterminacy in LAM, since they are also expressed in LP of determinate organs. The presence of other factors specific to the LAM region is presumed to regulate or support the functions of these genes.

It is noteworthy that indeterminate compound leaves may have evolved independently in *Guarea* and *Chisocheton*^25^, both genera of Meliaceae, and this type of indeterminate leaf has not been reported outside this family. This suggests that Meliaceae species share a preadaptive trait to acquire indeterminacy in LM. Meliaceae species typically have determinate pinnate leaves, and development of these is acropetal, with a meristem at the tip of the leaf. This meristem produces a protrusion that elongates prior to the maturation of the newest pair of leaflets, termed ‘vorläuferspitze’ meaning ‘forerunner tip’ in Germany^59^. In species with determinate leaves, vorläuferspitze finally differentiates into a terminal leaflet or is abscised^22^. On the other hand, in *Guarea* and *Chisocheton*, instead of forming vorläuferspitze, leaf-tip buds are retained for years. We therefore speculate that maintenance of a meristem for vorläuferspitze may be a preadaptive feature for indeterminacy in leaves in Meliaceae. Feature comparison between vorläuferspitze of a species with determinate leaves and leaf-tip buds of indeterminate leaves of *Guarea* and *Chisocheton* will provide insight into understanding the specific evolution of indeterminate pinnate leaves in Meliaceae, and the present work provides a basis for further studies.

## Materials and methods

### Plant materials

We used *Guarea glabla* Vahl plants grown in a greenhouse in the Botanical Gardens, Graduate School of Science, The University of Tokyo (Koishikawa, Tokyo, Japan). They were originally collected in Sierra de Luquillo, Puerto Rico, and were identified, propagated, used for the previous studies^22,24^ and provided by Dr. Jack Fisher of Fairchild Tropical Botanic Garden (Miami, USA). All experiments on this plant were carried out in accordance with relevant institutional, national, and international guidelines and legislation.

### RNA-seq

For RNA extraction, plant tissues were frozen in liquid nitrogen immediately after they were dissected from the tree. We collected leaf apex tissue from leaves in which several leaflet pairs were already expanded. Frozen tissues were homogenised using a TissuLyserII instrument (Qiagen, Venlo, Netherlands) with zirconia beads, and RNA was extracted using PureLink Plant RNA Reagent (Thermo Fisher Scientific, Waltham, Massachusetts, USA) following the supplied protocol. Because the quality of RNA following this step was not adequately high, we further purified it using AMpureXP beads (Beckman Coulter, Brea, California, USA), and confirmed that the obtained RNA was sufficiently pure (RNA Integrity Number (RIN) > 6) using a Bioanalyzer and an Agilent 2100 RNA6000 nano kit (Agilent Technologies, Santa Clara California, USA). For library preparation, we used a KAPA mRNA Hyper Kit (Roche, Basel, Switzerland) following the manufacturer’s protocol. We made libraries from three independent tissue samples as biological replicates. For transcriptome assembly, we mixed all libraries and performed sequencing with 150 bp paired-end reads using a HiseqX Ten platform (Illumina, San Diego, California, USA). For expression analyses, we sequenced libraries separately with 100 bp single-end reads using a Hiseq1500 platform (Illumina). Raw reads have been deposited in the DDBJ Sequence Read Archive (DRA) under BioProject PRJDB16673. Run IDs are listed in Supplemental Table S1.

### Transcriptome assembly and read quantification

Paired-end reads obtained from *G. glabra* shoot tissues were quality-trimmed by trimmomatic (version 0.36, minimum length: 32)^60^ and assembled by Trinity (version 2.8.1)^61^. Contigs derived from rRNA were identified by rnammer (version 1.2.1)^62^ and removed from the assembly. In addition, contigs that showed the best hit (e-value < 1e−50) to a sequence from non-Viridiplantae species in BLASTn results against the NT database were removed as possible contaminants. Single open reading frames (ORFs) of each transcript were predicted by Transdecoder (version 5.5.0)^63^ and the predicted coding sequences were annotated by their orthology using eggnog-mapper^64,65^.

Single-end sequences obtained from different tissues (three replicates) were quantified using the filtered transcriptome assembly as a reference by Salmon (version 0.14.1)^66^. Before gene expression patterning, counts were normalised by the TCC package (version 1.41.0) using edgeR (version 3.40.2)^67,68^. Posterior probabilities (PP) for all possible differential expression patterns were calculated by the baySeq package (version 1.31.0)^27^, and the pattern with the highest PP value was assigned to a gene. Gene Ontology (GO) enrichment analyses were performed by the GOstats package (version 2.66.0)^69^.

### Phylogenetic analyses

Using amino acid sequences of Arabidopsis KNOX proteins as queries, we retrieved sequences with high similarity using BLASTp against the customised database in which the *G. glabra* proteome and 67 public proteome datasets collected from Phytozome (https://phytozome-next.jgi.doe.gov/) and other sources^70,71^ were integrated (Table S2). Sequences were aligned with MAFFT (version 7.480)^72^, and possible non-homologous sites were trimmed from the alignment by TrimAL (version 1.4)^73^. Maximum likelihood trees were reconstructed by IQ-TREE (version 2.31.1)^74,75^.

### Gene cloning and RNA probe preparation

cDNA was reverse-transcribed from RNA employed for RNA-seq using a SuperScript III First-Strand Synthesis System for RT-PCR kit (Thermo Fisher Scientific). Next, using primers listed in Supplementary Table S3, PCR was performed to amplify the whole CDS region of the target gene using PrimeStar GXL DNA polymerase (Takara, Kyoto, Japan). Amplicons were subcloned into the pZErO-2 vector (Thermo Fisher Scientific). Digoxigenin (DIG)-labelled RNA probe was transcribed with T7 or SP6 RNA polymerase (Roche) from the PCR product amplified from the cloned plasmid using the M13 primer set.

### Technovit sectioning

Tissues for anatomical observations and ISH were dissected into pieces < 1 cm in length, followed by Formalin-Acetic acid-Alcohol (FAA) fixation for > 1 h. Fixed samples were dehydrated using a stepwise ethanol gradient to 99.5%, incubated at room temperature for 30 min in each step. Dehydrated samples were transferred to the appropriate Technovit resin. For simple tissue observation, Technovit 7100 or 8100 (Kulzer, Hanau, Germany) was used. After the substitution process, resin was hardened in Histform S (Kulzer) as described in the supplied protocol. Sections 7–10 µm thick were prepared using an HM360 microtome (Leica Biosystems, Wetzlar, Germany). Sections were stained with 0.1% Toluidine Blue O.

For ISH, Technovit 9100 (Kulzer) resin was used^31^. Before use, resin was destabilised by adding 10% (w/v) activated alumina, followed by vigorous shaking for 1 h. Before transferring resin, samples were incubated twice in xylene for 1 h each time to remove ethanol, then incubated in pre-infiltration solutions 3 and 2 for 1 h each. Samples were then treated with pre-infiltration solution 3 for 2 days at 4 °C. Samples were transferred to a mixture of polymerisation solutions A and B at a 9:1 ratio, degassed on ice, then polymerised at 4 °C for 3–5 days in Histform S (Kulzer). Using a HM360 microtome, Sections 20–30 µm thick were prepared and transferred into a microtube containing 50% ethanol. To remove the Technovit9100 resin, the sections were treated with 2-methoxyethyl acetate for 20 min at room temperature, then rehydrated in a 90%, 70% and 50% (v/v) ethanol series for 5 min each step. Finally, sections were transferred to pure RNase-free water to serve for ISH.

### Section ISH

Sections were attached to a MAS-coated glass slide (Matsunami Glass, Osaka, Japan) and washed with phosphate-buffered saline (PBS). For ISH, we essentially followed the protocol for whole-mount ISH applied for various plants^21,76,77^, which was modified for sections on a glass slide by removing the detergent from solutions.

### Supplementary Information

Supplementary Figures.

Dataset S1.

Dataset S2.

Supplementary Tables.

## Data Availability

Sequence raw data was deposited to DDBJ/EBI/NCBI (Supplemental Table S1). Transcriptome assembly, expression data, gene annotations, and alignment data for phylogenetic analyses are available in figshare (10.6084/m9.figshare.24354829). All other data generated or analysed during this study are included in this published article and its Supplementary Information files.
