# Supplementary material for: Decoding the leaf apical meristem of Guarea glabra Vahl (Meliaceae): insight into the evolution of indeterminate pinnate leaves
Source: Sci Rep. 2024 Mar 2;14:5166. doi: 10.1038/s41598-024-55882-0 (PMC10908829; doi:10.1038/s41598-024-55882-0)
Supplement: Supplementary file 1 — Supplementary Figures. [file 41598_2024_55882_MOESM1_ESM.pdf]

Supplementary Information

Decoding the Leaf Apical Meristem of *Guarea glabra* Vahl (Meliaceae): Insight into the Evolution of Indeterminate Pinnate Leaves

Yasutake Moriyama, Hiroyuki Koga, Hirokazu Tsukaya

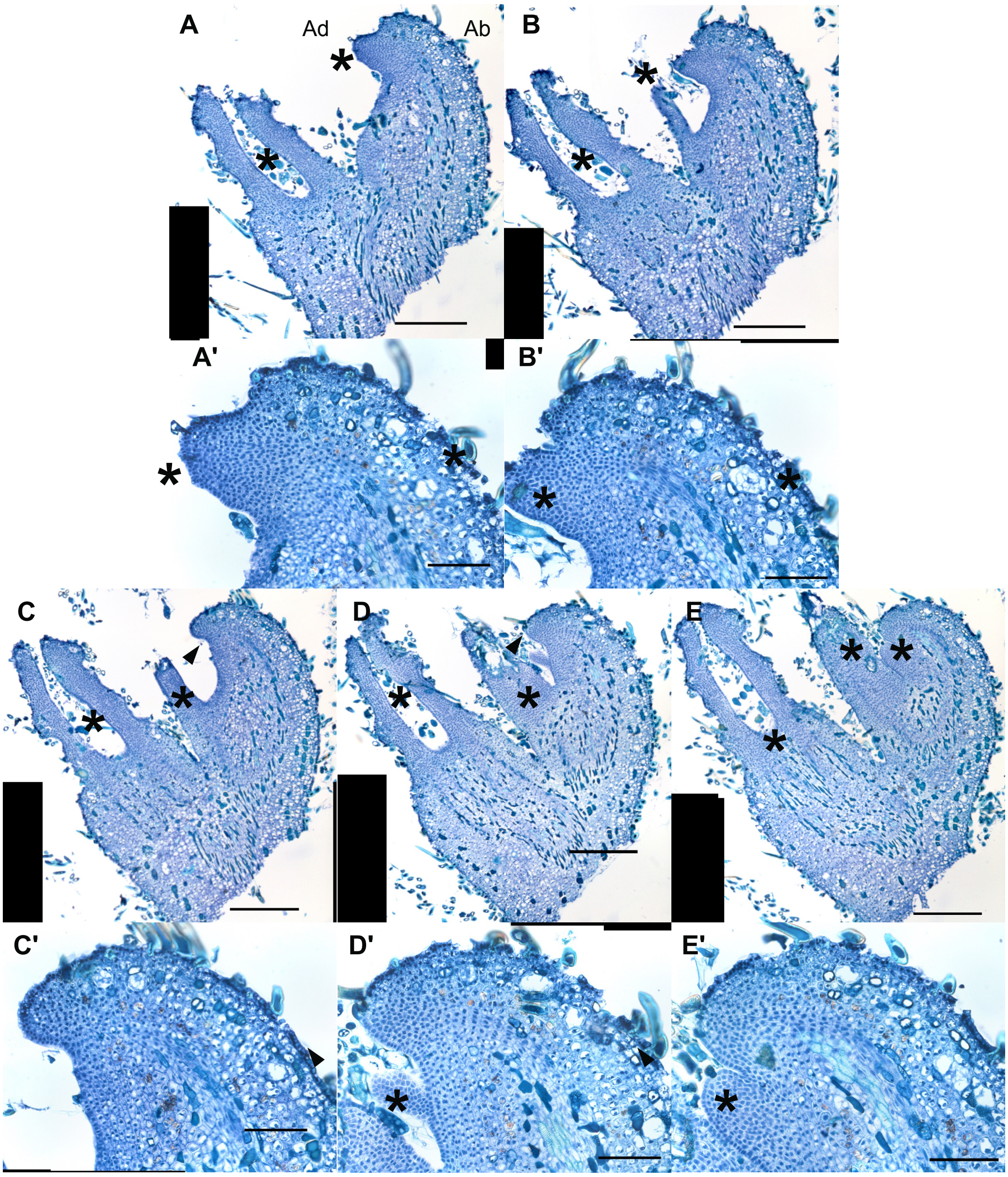

Supplementary Figure S1: Serial sections of a leaf apex of *G. glabra*.

(A–E) Sections were sliced every 20 µm in the order A to E. (A'–E') Magnified images of leaf apices. Asterisks indicate leaf primordia and arrowheads indicate leaf apical meristem. Ab, abaxial side; Ad, Adaxial side; Scale bars A–E = 300 µm and A'–E' =100

µm.

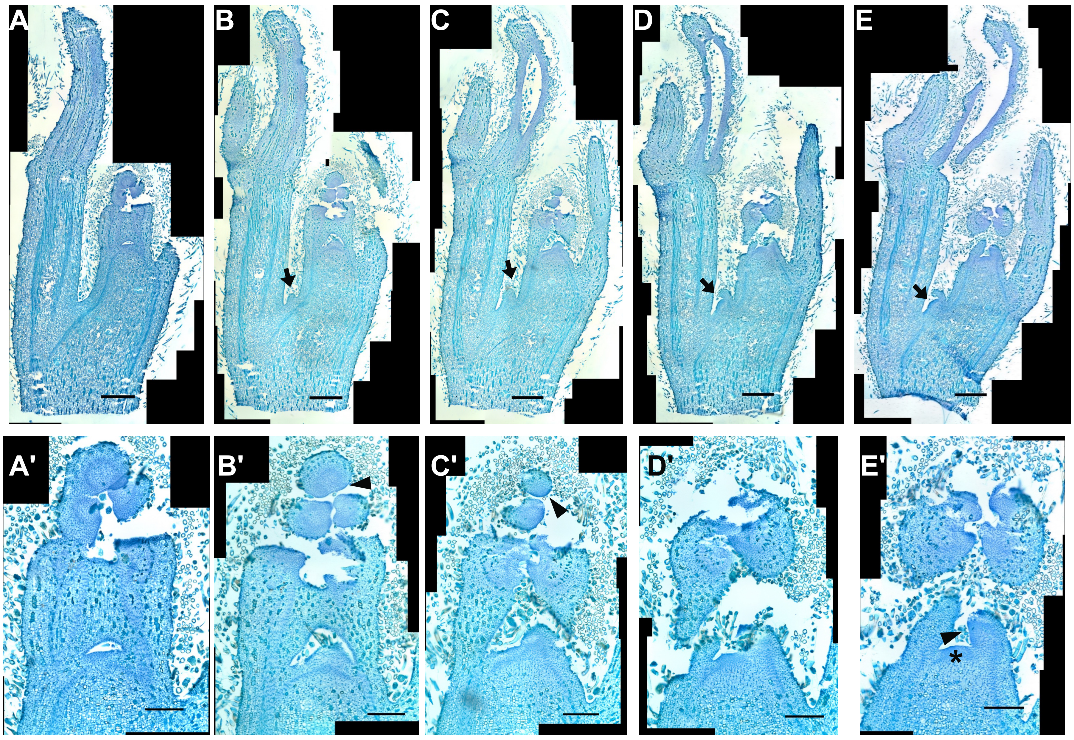

Supplementary Figure S2: Serial sections of a shoot apex of *G. glabra*.

Sections were sliced every 20 µm in the order A to E. (A'–E') Magnified images around the shoot apex. Asterisks indicate shoot apical meristem, arrowheads indicate leaf apical meristem, and arrows indicate axillary buds. Scale bars A–E = 500 µm and A'–E' = 200 µm.

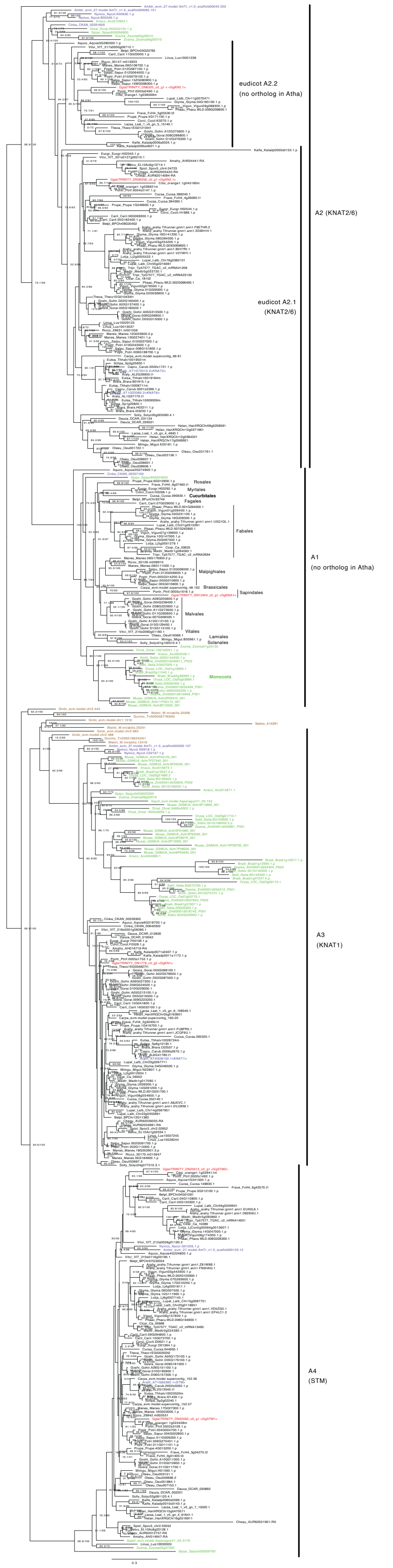
eudicot A2.2
(no ortholog in Atha)
A2 (KNAT2/6)
eudicot A2.1
(KNAT2/6)
Rosales
Myrtales
Cucurbitales
Fagales
Fabales
Malpighiales
Brassicales
Sapindales
Malvales
Vitales
Lamiales
Solanales
Monocots
A1
(no ortholog in Atha)
A3
(KNAT1)
A4
(STM)

Supplemental Figure S3: Maximum likelihood tree of KNOX1 proteins.

This is the complete version of the tree in Figure 2C. Node values represent Shimodaira-Hasegawa-like approximate likelihood ratio test (SH-aLRT) support (%)/ultrafast bootstrap (UFboot) support (%).

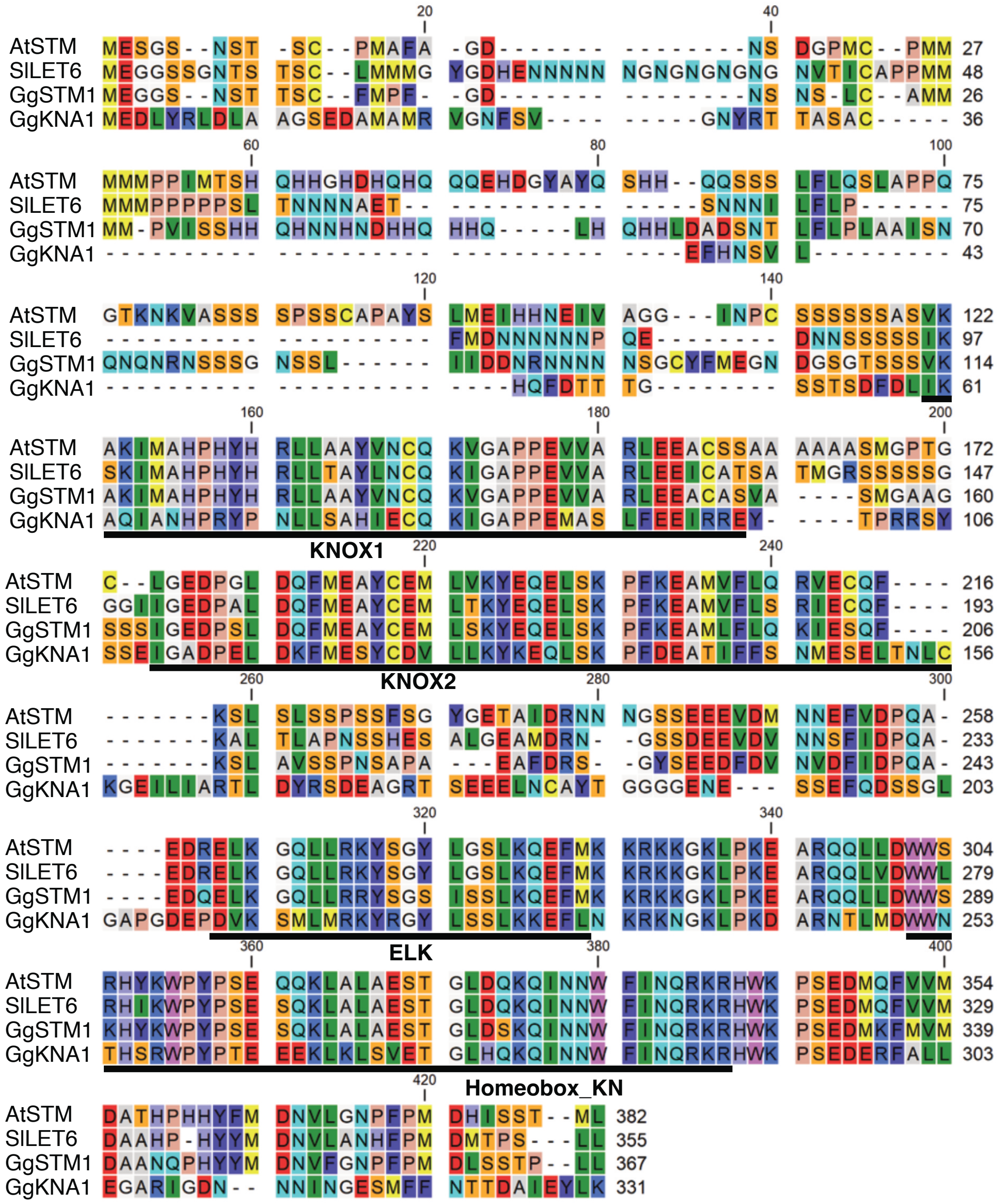

Supplemental Figure S4: **Amino acid sequence alignment of GgKNA1 and other KNOX1 proteins.**

Four domains characteristic of the KNOX1 protein (KNOX1, KNOX2, ELK and Homeobox_KN) are conserved. Underlines indicate the pfam domains identified in GgKNA1 by interproscan. At, *A. thaliana*; Gg, *G. glabra*; Sl, *S. lycopersicum* (tomato).

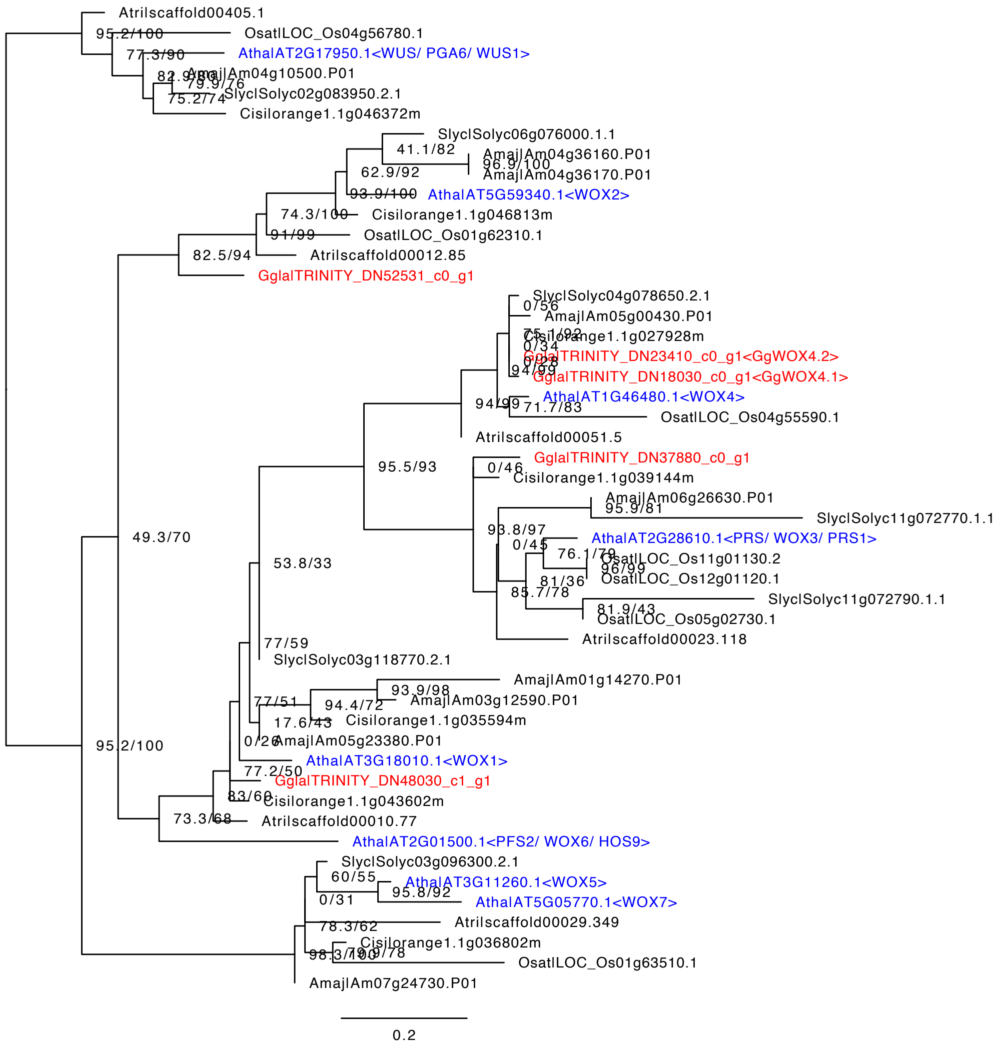

Supplementary Figure S5: WOX protein phylogenetic tree

A maximum likelihood tree is shown. Node values represent SH-aLRT support (%)/UFboot support (%). Amaj, *Antirrhinu majus*; Atha (blue), *Arabidopsis thaliana*; Atri, *Amborella trichopoda*; Cisi, *Citrus sinensis*; Ggla (red), *G. glabra*; Gmax, *Glycine max*; Grai, *Gossypium raimondii*; Slyc, *Solanum lycopersicum*; Osat, *Oriza sativa*.

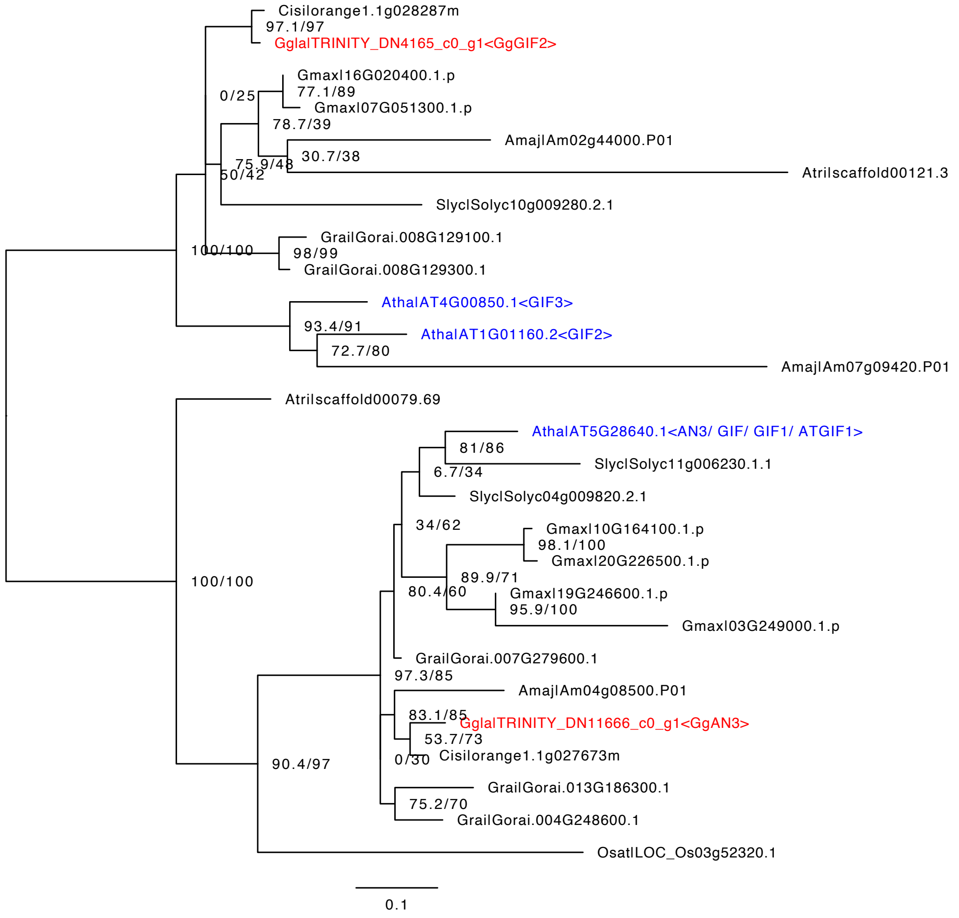

Supplementary Figure S6: GIF protein phylogenetic tree.

A maximum-Likelihood tree is shown. Node values represent SH-aLRT support (%)/UFboot support (%). Amaj, *Antirrhinu majus*; Atha, *Arabidopsis thaliana*; Atri, *Amborella trichopoda*; Cisi, *Citrus sinensis*; Ggla, *G. glabra*; Gmax, *Glycine max*; Grai, *Gossypium raimondii*; Slyc, *Solanum lycopersicum*; Osat, *Oriza sativa*.

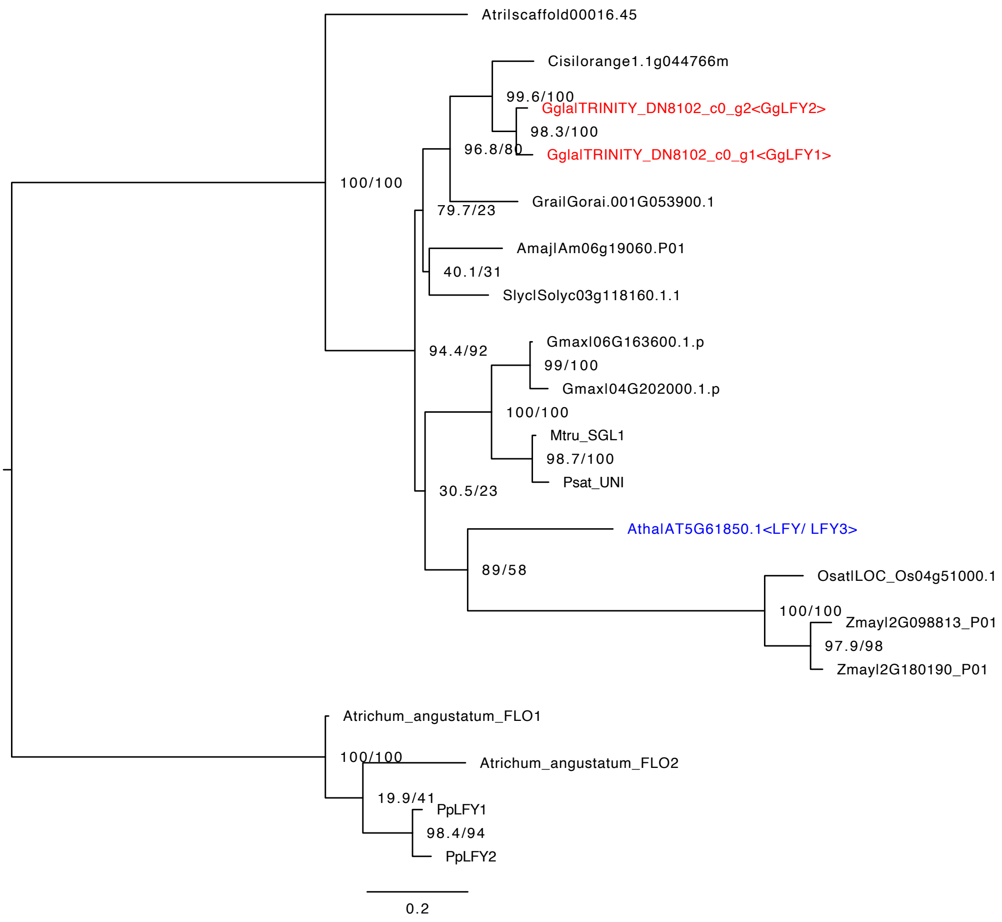

Supplementary Figure S7: LFY/FLO protein phylogenetic tree.

A maximum likelihood tree is shown. Node values represent SH-aLRT support (%)/UFboot support (%). Amaj, *Antirrhinu majus*; Atha, *Arabidopsis thaliana*; Atri, *Amborella trichopoda*; Cisi, *Citrus sinensis*; Ggla, *G. glabra*; Gmax, *Glycine max*; Grai, *Gossypium raimondii*; Mtru, Medicago *truncatula*; Pp, *Physcomitruum patens*; Psat, *Pisum sativum*; Slyc, *Solanum lycopersicum*; Zmay, *Zea mays*.
